# Improving Light Stability of Nonfullerene Acceptor Inverted Organic Solar Cell by Incorporating a Mixed Nanocomposite Metal Oxide Electron Transporting Layer

**DOI:** 10.1021/acsaelm.5c00201

**Published:** 2025-04-24

**Authors:** Apostolos Ioakeimidis, Fedros Galatopoulos, Alina Hauser, Michael Rossier, Stelios A. Choulis

**Affiliations:** † Molecular Electronics and Photonics Research Unit, Department of Mechanical Engineering and Materials Science and Engineering, Cyprus University of Technology, Limassol 3603, Cyprus; ‡ Avantama AG, Laubisruetistr. 50, Staefa 8712, Switzerland

**Keywords:** organic photovoltaics, metal oxides, electron transporting layers, lifetime, inverted organic solar cells

## Abstract

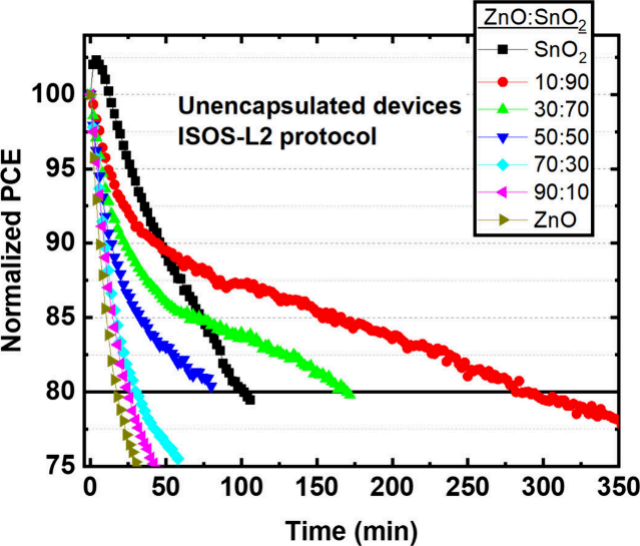

We present significant light stability enhancement of nonfullerene acceptor inverted organic photovoltaics by incorporating a mixed nanocomposite metal oxide electron transporting layer. Using an appropriate mixture of ZnO:SnO_2_ nanoparticles as an electron transporting layer in a PBDB-TF-T1 (T1):IT4F based organic solar cell device mitigates light induced photodegradation by lowering the defect formation at the active layer interface. We propose that the mixed metal oxide ETL act as hole scavengers that reduces the photocatalytic reaction of its surface. The optimized nanocomposite mixture of ZnO:SnO_2_ 10:90 (%V) provides higher light stability (ISOS-L2 protocol), prolonging the inverted OSCs lifetime (80% of the initial PCE, T80) by ∼16.5 times compared to the commonly used pristine ZnO electron transporting layer.

## Introduction

Organic solar cells (OSCs) constitute one of the most promising next-generation photovoltaic technologies for implementation in a vast number of applications. The intense research and development in terms of materials and devices have resulted in an increase of OSC power conversion efficiency (PCE), reaching the milestone of ∼20%.^1,2^ While PCE is one prerequisite toward organic photovoltaics commercialization, additional challenges need to be addressed. The most impactful limitation is the long-term stability of OSC hindering their widespread adoption.^3^ The various degradation mechanisms occur within the bulk of the device’s functional layers as well as at their interfaces due to stressing factors including oxygen, moisture, high temperature, and UV content of light.^4−6^ Implementation of sophisticated materials and processes have demonstrated the potential of extending the operating lifetime under controlled conditions to thousands of hours.^7−10^ Nevertheless, additional factors that impact the commercialization of OPVs must be taken into consideration such as raw materials cost, synthesis and processing complexity, toxicity, etc.^11−14^

The material properties of common metal oxides such as the high optical band gap, stability, facile energy levels, and electrical tunability in combination with nature abundant raw materials, easy synthesis, and low-cost processing render them a strong candidate for charge transporting layers in commercial OPVs. Regarding the so-called inverted structure, which is the commercially preferred structure for large scale production, the commonly used electron transporting layers (ETLs) and hole transporting layers (HTLs) are ZnO and MoO_3_, respectively. Beyond these common metal oxides, a variety of pristine and modified metal oxides have been successfully applied as ETLs and HTLs, resulting in improved PCE and lifetime.^15−19^ Nevertheless, the proven photocatalytic reactivity of front contact metal oxides can have a detrimental effect on the stability of OSCs through degradation of the active layer. Passivation of metal oxide photocatalytic sites at the interface with the active layer is a common strategy to alleviate surface reactivity.^20−24^ Jiang et al. demonstrated that an SnO_2_ ETL has a reduced photocatalytic effect on the IT4F improving the light stability of OPV compared to ZnO.^25^ Recently, Park et al. improved the photostability of a nonfullerene acceptor (NFA) OPV by replacing the common ZnO ETL with a UV-A-insensitive titanium suboxide layer that suppressed the photocatalytic effect.^26^

In this manuscript we demonstrate a facile and scalable strategy to improve the OSC light stability implementing a mixed nanocomposite metal oxide ZnO:SnO_2_ ETL. We study a full scale ratio of ZnO:SnO_2_ mixed metal oxide nanoparticles (NPs) as ETLs using ITF4 as the active layer acceptor ,which is very sensitive to the photocatalytic effect of the ZnO layer.^25^ The implementation of a 10:90 ratio ZnO:SnO_2_ NP ETL in an inverted device structure results in ∼16.5 and ∼2.8 times improved light stability (ISOS-L2 protocol) compared to pristine ZnO and SnO_2_, respectively. The produced energy until 80% of the initial power conversion efficiency (PCE) for the 10:90 ratio ZnO:SnO_2_ is about ∼13.2 and ∼3.0 times higher compared to the corresponding ZnO and SnO_2_ based devices, respectively. Electroimpedance and electrical measurements show that the light stability improvement can be attributed to the lower defect formation at the active layer interface with the metal oxide for the 10:90 ratio ZnO:SnO_2_ ETL compared to the pristine ZnO, SnO_2_, or other ratios of ZnO:SnO_2_ ETL.

## Materials and Methods

### Materials

Prepatterned glass-ITO substrates T1 (PBDB-TF-T1) and IT-4F were purchased from Ossila Ltd. The stannic oxide (SnO_2_ N-31) and zinc oxide (ZnO N-10-Flex) in a mixture of butanols (2.5 wt %) were supplied by Avantama AG. Silver (Ag) pellets (99.99%) were purchased from Kurt J. Lesker Company. All of the other chemicals in this study were purchased from Sigma-Aldrich.

### Device Fabrication

The ITO/glass substrates were sequentially sonicated in acetone and 2-propanol for 10 min and dried with N_2_ before use. The mixed metal oxides and the pristine ZnO and SnO_2_ metal oxide nanoparticle inks were blade coated at 70 °C on top of on ITO/glass substrates in air without any further treatment, resulting in ∼30 nm thick ETLs. The active layer solution T1:IT4F (1:1.2 wt %) dissolved in *o*-xylene were blade coated at 70 °C on top of the various underlayers in air and annealed at 100 °C for 10 min in a glovebox. *o*-Xylene was selected as a less toxic organic solvent compared to commonly used halogenated solvents (e.g., chlorobenzene), rendering the process more compatible for industrial large-scale fabrication. To complete the devices 10 nm MoO_3_ and 100 nm Ag were thermally evaporated resulting in a 4 mm^2^ active area.

### Characterization

The thickness of the films was measured with a Veeco Dektak 150 profilometer. AFM images were obtained using a Nanosurf easy scan 2 controller in tapping mode. For the current–voltage (*J*–*V*) characterization a 1 sun calibrated (100 mW/cm^2^, Oriel 91150 V calibration cell) Newport Solar simulator equipped with a Xe lamp was used. The *J*–*V* curves were obtained by using an Ossila Solar Cell I-V Test System. To extract the series resistance (*R*_s_) and shunt resistance (*R*_sh_) the slope of the light *J*–*V* curve was calculated at the region close to the *V*_oc_ and *J*_s_, respectively. The light stability of the unencapsulated devices was determined following ISOS-L2, where according to the protocol the measuring conditions are light source from solar simulator, device temperature 65 or 85 °C, ambient environment, and load at MPP or open circuit (*V*_oc_).^27^ In our case, the unencapsulated devices were exposed to 100 mW/cm^2^ solar simulated light in an ambient environment (RH ≈ 50%, temp ≈ 23 °C) while the devices were heated at ∼65 °C during the measurement. Each device was *J*–*V* scanned between −0.5 and 1.2 V every 2 min, and between it was kept at open circuit conditions. The extraction of the total energy produced by each sample was calculated by intergrating the power obtained by each sequential measurement until it reached 80% of the initial PCE. The absorption measurements were performed with a Shimadzu UV-2700 UV–vis optical spectrophotometer. Electroimpedance spectroscopy (EIS) measurements were obtained using a Metrohm Autolab PGSTAT 302N. Mott–Schottky measurement was obtained by applying a small AC perturbation of 10 mV, a frequency of 1000 Hz, and dark conditions. −*Z*″-Frequency measurement was obtained by applying a small AC perturbation of 10 mV at 0.9 V bias and under dark conditions.

## Results and Discussion

Inverted OSCs with the structure ITO/ZnO:SnO_2_ mixture ETL/PBDB-TF-T1 (T1):IT4F/MoO_3_/Ag were fabricated applying a full range of ZnO:SnO_2_ ratios as ETLs. Specifically, for the preparation of the various mixed ETLs, ZnO and SnO_2_ metal oxide nanoparticle inks were mixed in volume ratios 10:90, 30:70, 50:50, 70:30, and 90:10. Each batch of inverted OSCs incorporating mixed metal oxide or pristine metal oxide ETLs consists of 16 different devices.

Figure S1 depicts the *J*–*V* curves of the best obtained PCE for each device structure while the corresponding PV parameters as well as the mean PV parameters with the standard deviation in parentheses are shown in Table 1 (Figure S2a–d as graph). It is seen that increasing the ZnO ratio in the mixed metal oxide ETL leads to a higher mean PCE, with the highest mean PCE (10.73%) obtained for pristine ZnO ETL. Regarding the PV parameters, ratios of ZnO above 50% increases the mean *V*_oc_ from 0.94 to 0.96 V. The mean *J*_sc_ and FF also show an increasing trend for higher ratios of ZnO within the ETL. The lower PCE of the SnO_2_ compared to ZnO based devices is ascribed to the higher energy offset of the LUMO energy levels between the IT4F (−4.10 eV)/SnO_2_ (−4.5 eV) compared to IT4F (−4.10)/ZnO (−4.3 eV) (literature reported values) resulting in *V*_oc_ losses.^28−30^ Additionally, the higher mean series resistances (*R*_s_) (Figure S2e) and the lower shunt resistance (*R*_sh_) (Figure S2f) result in lower FF and *J*_sc_.

**Table 1:** Best PCE Devices under AM1.5 G Light Incorporating Various Ratios of Mixed ZnO:SnO_2_ NP ETL with Their Corresponding *V*_oc_, *J*_sc_, and FF Parameters^a^

ZnO:SnO_2_ (%V)	*V*_oc_ (V)	Jsc (mA/cm^2^)	FF (%)	PCE (%)
0:100	0.94 (0.94 ± <0.005)	15.37 (15.39 ± 0.39)	57.21 (55.99 ± 0.82)	8.27 (8.10 ± 0.17)
10:90	0.94 (0.94 ± <0.005)	16.69 (16.19 ± 0.46)	59.00 (58.67 ± 0.47)	9.26 (8.94 ± 0.35)
30:70	0.94 (0.94 ± < 0.005)	16.86 (16.43 ± 0.37)	59.84 (59.91 ± 0.46)	9.49 (9.27 ± 0.23)
50:50	0.94 (0.94 ± <0.005)	16.71 (16.40 ± 0.34)	61.23 (61.00 ± 0.33)	9.62 (9.43 ± 0.16)
70:30	0.96 (0.96 ± <0.005)	17.07 (16.49 ± 0.47)	60.5 (61.31 ± 0.75)	9.92 (9.67 ± 0.33)
90:10	0.96 (0.96 ± <0.005)	17.17 (16.82 ± 0.24)	63.15 (63.15 ± 0.24)	10.41 (10.16 ± 0.15)
100:0	0.96 (0.96 ± <0.005)	17.85 (17.27 ± 0.67)	62.62 (61.95 ± 1.07)	10.73 (10.22 ± 0.50)

a The mean values and standard deviations obtained out of 16 different devices for each batch are given in parentheses.

**Figure 1:**
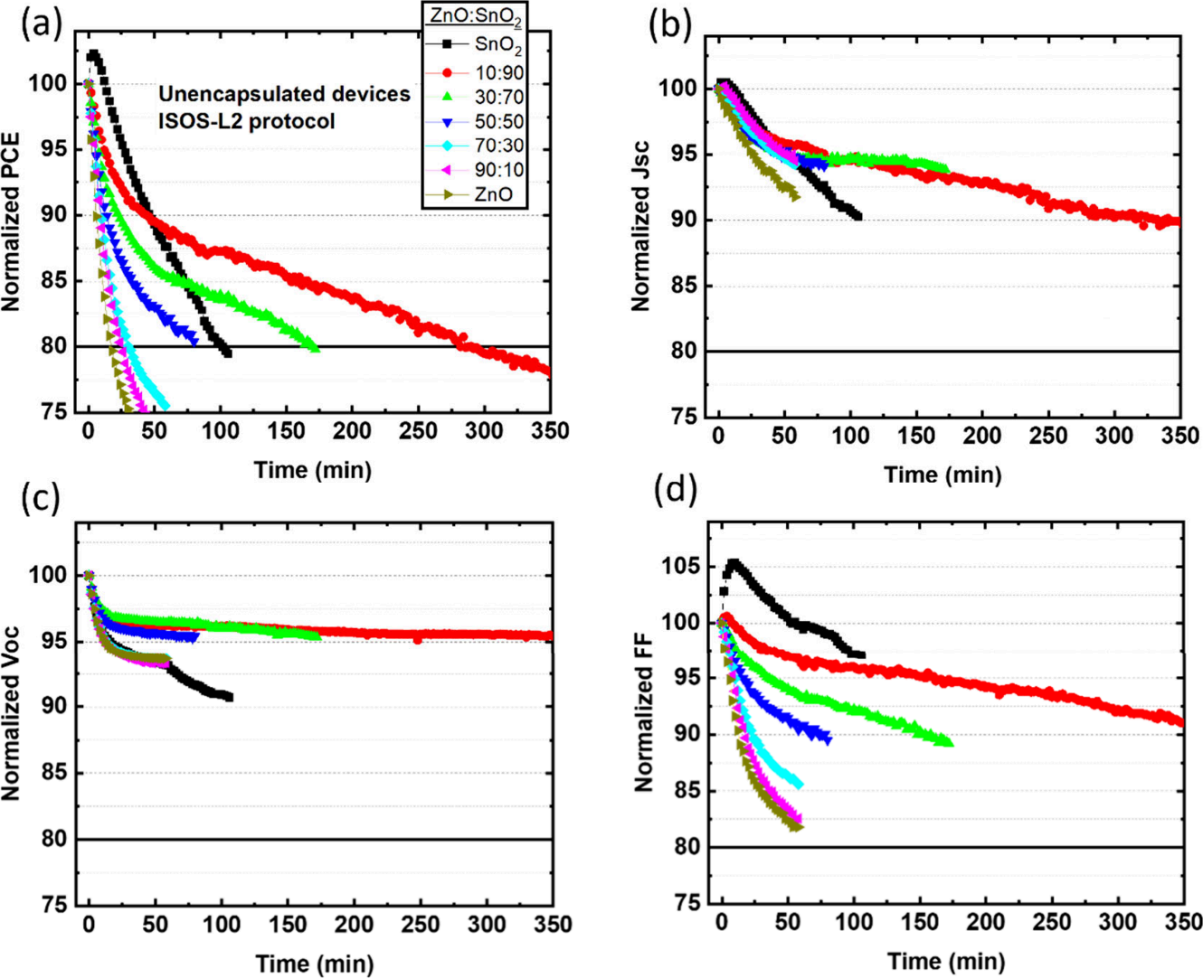
Normalized (a) PCE, (b) *J*_sc_, (c) *V*_oc_, and (d) FF of unencapsulated T1:IT4F based inverted OSC incorporating various ratios of mixed oxide ZnO:SnO_2_ ETLs as a function of exposure time under the ISOS-L2 protocol.

The unencapsulated devices were tested under the ISOS-L2 protocol to examine the stability of the devices. Figure 1a–d shows the normalized PCE, *J*_sc_, *V*_oc_, and FF with respect to light exposure time for the devices incorporating various ratios of mixed ZnO:SnO_2_ ETLs. The ZnO:SnO_2_ 10:90 mixture demonstrated the highest stability, prolonging the lifetime (80% of the initial PCE, *T*_80_) by ∼16.5 times compared to ZnO and by ∼2.8 times compared to SnO_2_. Specifically, the pristine SnO_2_ based devices reached *T*80 within ∼100 min after a light soaking increase of the initial PCE ascribed to photoinduced passivation of the surface defects.^31^ The devices based on ZnO:SnO_2_ ratios 10:90, 30:70, and 50:50 reached *T*_80_ after ∼280, ∼150, and ∼80 min, respectively. On the other hand, when the ZnO content within the ETL was above 50% *T*_80_ was reached after only 17–30 min. To estimate the trade-off between initial PCE and lifetime, we calculated the produced energy until *T*_80_ (Table 2). The highest electrical energy (1434 μWh/cm^2^) was provided by the OSC incorporating the ZnO:SnO_2_ ratio 10:90 ETL. The produced energy is about ∼13.2 and ∼3.0 times higher compared to the corresponding ZnO and SnO_2_ based OSCs. Thus, the lower PCE of the 10:90 ZnO:SnO_2_ based device compared to devices with higher ratios of ZnO content is compensated by the improved lifetime, resulting in more efficient devices. Figure S3 shows the *T*_80_ lifetime (left axis) and the corresponding calculated produced energy (right axis) for the OSC based on the various ratios of ZnO:SnO_2_ ETL, visualizing the strong correlation between these two parameters.

**Table 2:** Electrical Energy Produced under ISOS-L2 Protocol until *T*_80_ by the Unencapsulated T1:IT4F Based Inverted OSC Incorporating Various Ratios of Mixed ZnO:SnO_2_ ETL

ZnO:SnO_2_ (%V)	*E* (μWh/cm^2^)
0:100	480
10:90	1434
30:70	908
50:50	442
70:30	181
90:10	156
100:0	109

Further investigation was conducted for a deeper understanding of the lifetime enhancement of the OSC incorporating ZnO:SnO_2_ 10:90. The surface topography of the various films fabricated on top of indium tin oxide (ITO) substrates was obtained using AFM. Figure S4a,g presents the obtained topography images (10 μm × 10 μm) of the various metal oxide films applying the same processing parameters used for the fabrication of the corresponding OSC. The extracted roughness (RMS) of the films demonstrates negligible difference between the various films ranging from 3.7 to 4.6 nm. From the corresponding AFM phase images (Figure S5a–g) we can infer that all the films under study exhibit pinhole-free films based on the observation of the smooth frequency images. Thus, we rule out that such small morphological differences of the ETL films affect the lifetime performances of the OSC under study.

**Figure 2:**
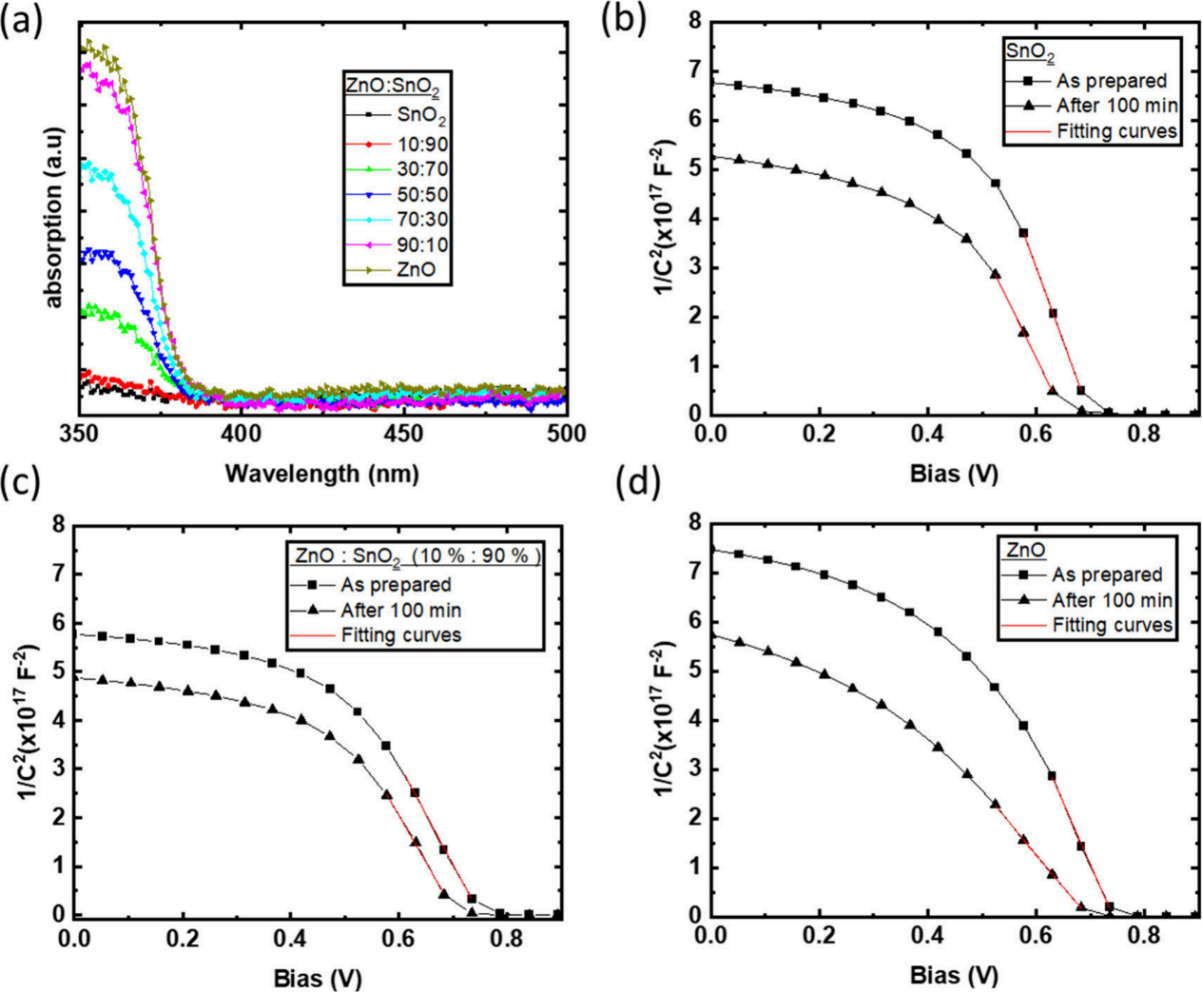
(a) UV–vis absorption graph of mixed oxide ZnO:SnO_2_ ETL and Mott–Schottky plots of unencapsulated T1:IT4F based inverted OSCs incorporating (b) SnO_2_, (c) mixed oxide ZnO:SnO_2_ 10:90 ETL, and (d) ZnO for the as-prepared devices and after 100 min under the ISOS-L2 protocol.

Figure 2a shows the UV–vis absorption graph of various mixed metal oxide ZnO:SnO_2_ ETLs fabricated on top of the glass substrates. The films exhibit an increasing absorption coefficient at wavelengths shorter than 400 nm for higher ratios of ZnO within the ZnO:SnO_2_ ETL. Thus, illumination with UV containing light will result in a higher number of photogenerated electron–hole pairs in the metal oxide ETL, which in turn increases the photoreactivity of the layers. On the other hand, SnO_2_ has a higher band gap and thus lower absorption coefficient at the near UV region resulting in lower electron–hole pair photogeneration, rendering the SnO_2_ less photoreactive. However, a marginal degradation of the interface results in the formation of energetic barriers and trap states that impedes the efficient charge transfer to the contacts.^32^ Thus, and given the light stability measurement shown above, pristine SnO_2_ can degrade the very delicate metal oxide/active layer interface. The addition of 10% ZnO NP in SnO_2_ marginally affects the absorption at wavelengths shorter than 400 nm. Thus, we rule out that the difference in lifetime between pristine and 10% ZnO based devices is affected by UV absorption properties.

Figure 2b–d and Figure S6a–d present the Mott–Schottky plots of unencapsulated T1:IT4F based inverted OSCs incorporating the various ratios of mixed metal oxide ZnO:SnO_2_ ETLs on the as-prepared unencapsulated inverted OSC and after 100 min under the ISOS-L2 protocol. Mott–Schottky plots can provide information about the doping profile of the semiconductor through the modulation of the depletion zone according to the relation
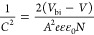
where *N* is the doping density.^33,34^ Thus, we can get an estimation for the light induced formation of a defect state that can act as dopants close to the interface by calculating the slope in the Mott–Schottky plot at bias close to *V*_bi_ before and after degradation. The increases in slope (order of magnitude 10^17^) before and after the illumination are 0.77 and 1.27 for the SnO_2_ and ZnO ETL based inverted OSC, respectively. In contrast, the ZnO:SnO_2_ 10:90 ETL based device exhibits the lowest slope increase by only 0.13, pointing to lower ETL induced defect formation close to the interface. The calculated slopes for the 30:70, 50:50, 70:30, and 90:10 ETL based devices increase by 0.49, 0.55, 1.30, and 1.29, respectively.

**Figure 3:**
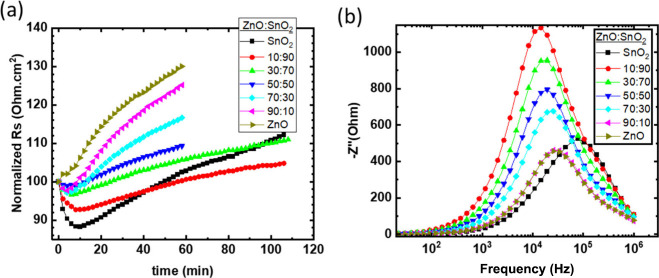
(a) Normalized series resistance of unencapsulated T1:IT4F based inverted OSCs incorporating various ratios of mixed oxide ZnO:SnO_2_ ETL under ISOS-L2 and (b) the −*Z*″–frequency of the as-preparaed device.

We also calculated the relative change of *R*_s_ from the light *J*–*V* curves with respect to the time (Figure 3a). The *R*_s_ increase can be ascribed to the increase of the electron extraction barrier (higher contact resistance) due to the widening of energetic mismatch of the metal oxide/IT4F interface and the reduced electron mobility induced by the formed defects.^32^ The ZnO:SnO_2_ 10:90 ETL based inverted OSC shows the lowest *R*_s_ after 100 min illumination compared to any other ratio of ZnO:SnO_2_ in accordance with the Mott–Schottky results. Impedance (−*Z*″)–frequency measurements were performed to gain a better insight into the function of the ZnO:SnO_2_ 10:90 based inverted OSCs. Figure 3b) shows the −*Z*″–frequency spectra of the as-prepared devices. Each peak represents a characteristic relaxation process, where the peak frequency (*f*_p_) corresponds to the relaxation time (τ) of the process through the inverse relationship τ = *f*^–1^/2π. The ZnO or SnO_2_ based inverted OSCs exhibit a characteristic *f*_p_ for each device. The inverted OSC incorporating ZnO:SnO_2_ 10:90 clearly shows an overlap of at least two peak frequencies that can be reproduced by the SnO_2_ based device *f*_p_ and a new peak *f*_p_ with 1 order of magnitude lower frequency. Thus, an additional relaxation process is present with 1 order of magnitude higher τ. Considering that the content of ZnO nanoparticles in the ZnO:SnO_2_ 10:90 based ETL is quite low, the vast majority of ZnO nanoparticles are dispersed within the SnO_2_ nanoparticle matrix, and thus a negligible number of ZnO NPs are in physical contact with the active layer. Thus, we exclude that this new peak originates from a detectable charge transfer process at the ZnO:SnO_2_ (10:90)/active layer interface between the ZnO NPs and the active layer but rather signifies a charge transfer process at the SnO_2_/ZnO heterojunction. Based on the above, we argue that under working conditions and due to the ZnO/SnO_2_ heterojunction the UV light photogenerated holes in SnO_2_ are transferred to the ZnO HOMO (lower HOMO level of SnO_2_ compared to ZnO). The holes, which would otherwise be transferred to the metal-oxide/active layer interface are now trapped in ZnO nanoparticles due to the potential well. When a higher concentration of ZnO nanoparticles is present in the ZnO/SnO_2_ ETL, the higher surface area of the ZnO NPs results in physical contact between the ZnO NPs and the active layer, promoting the hole charges to the metal oxide/active layer interface. The trapped holes (minority carriers) will be annihilated by the electrons (majority carriers) through recombination, inducing a reduction of the *R*_sh_. This argument is in accordance with the findings presented in Figure S2f, where for ZnO:SnO_2_ 10:90 the devices exhibit the lowest mean *R*_sh_ due to the highest number of trapped holes, as discussed above. On the other hand, the photogenerated valence electrons in SnO_2_ experience an energetic barrier that blocks their easy transfer from SnO_2_ to ZnO, while the photogenerated electrons in ZnO easily drift to SnO_2_ due to lower LUMO levels of SnO_2_ (higher electron affinity).

A suggested degradation mechanism is attributed to the photogenerated electron hole pair in the metal oxide that consists of reductive and oxidative species, respectively. These photogenerated charges in the metal oxide ETL can oxidize or reduce directly or indirectly the organic molecules of the active layer at the interface, resulting in device performance degradation.^28,35,36^ Under working conditions, most of the electron charges drift and are collected by the metal contact, while the generated holes close to the interface with the active layer can drift/diffuse to the metal oxide/active layer interface. These holes can oxidize the surface absorbed water or hydroxyl groups, producing hydroxyl radicals (^•^OH).^37^ The reactivity of the metal oxide occurs predominantly on the surface of the ETL, resulting in the oxidation of the active layer inducing impurities near the surface of the active layer.^38^ The addition of a low density of ZnO NPs within the SnO_2_ nanoparticle ETL result in the dispersion of the ZnO NPs in the bulk SnO_2_. This composite structure drifts and traps the generated holes in the potential well formed by the SnO_2_/ZnO contact, as shown by the −*Z*″–frequency measurements, restraining the number of holes that can reach the ETL surface. Thus, the defect formation is reduced at the metal oxide/active layer interface, which agrees with the Mott–Schottky data and lower increase of the device *R*_s_ as a function of exposure time. A schematic illustration of the above-described process is presented in Figure S7.

Thus, we propose that the dispersion of 10% ZnO nanoparticles within the SnO_2_ nanoparticles can act as hole scavengers that reduce the photocatalytic reaction of the metal oxide based ETL surface leading to improved stability of the OSC under the ISOS-L2 protocol.

## Conclusion

In conclusion, we demonstrate the fabrication of inverted OSCs based on the binary active layer system PBDB-TF-T1 (T1):IT4F using a full range of ZnO:SnO_2_ NP mixtures as ETLs. Unencapsulated solar cell devices incorporating 10:90 ZnO:SnO_2_, even though they provided a lower PCE, the total produced power until reaching 80% of the initial efficiency is about ∼13.2 and ∼3.0 times higher compared to the corresponding ZnO and SnO_2_ based inverted OSCs. This results from improved stability (ISOS-L2 protocol) that prolongs lifetime (80% of the initial PCE, *T*_80_) by ∼16.5 times compared to ZnO and by ∼2.8 times compared to SnO_2_.The lifetime improvement for 10:90 ZnO:SnO_2_ is attributed to lower impurity formation at the active layer interface with the metal oxide. Specifically, we propose that the dispersion of 10% ZnO NPs within the SnO_2_ NPs acts as photogenerated hole scavengers, resulting in a reduced photocatalytic reactivity that mitigates active layer interface degradation.

## Supporting Information Available

*J*–*V* curves of the highest PCE devices under 1 sun simulated light (AM 1.5) of the unencapsulated inverted T1:IT4F based OSCs incorporating various ratios of mixed oxide ZnO:SnO_2_ ETLs, mean PCE, *J*_sc_, *V*_oc_, FF, *R*_s_, and *R*_sh_ under the ISOS-L2 protocol of the unencapsulated inverted T1:IT4F based OSCs incorporating various ratios of mixed oxide ZnO:SnO_2_ ETLs, *T*_80_ lifetimes (left axis) and the corresponding calculated produced energies (right axis) of the unencapsulated T1:IT4F based inverted OSCs incorporating various ratios of mixed oxide ZnO:SnO_2_ ETLs, AFM topography images (10 × 10 μm) of SnO_2_, 10:90, 30:70, 50:50, 70:30, and 90:10 ZnO/SnO_2_, and ZnO ETLs fabricated on top of ITO, AFM phase images (10 × 10 μm) of SnO_2_, 10:90, 30:70, 50:50, 70:30, and 90:10 ZnO:SnO_2_, and ZnO ETLs fabricated on top of ITO, Mott–Schottky plots of unencapsulated T1:IT4F based inverted OSCs incorporating mixed metal oxide ZnO:SnO_2_ 30:70, 50:50, 70:30, and 90:10 ETLs for the as-prepared and after 100 min ISOS-L2 protocol, and schematic illustration of the restrained photooxidation at the mixed metal oxide/active layer interface due to the presence of a low density of ZnO nanoparticles within the SnO_2_ ETL (PDF)
