# Supplementary material for: Improving Light Stability of Nonfullerene Acceptor Inverted Organic Solar Cell by Incorporating a Mixed Nanocomposite Metal Oxide Electron Transporting Layer
Source: ACS Appl Electron Mater. 2025 Apr 24;7(9):3940–6. doi: 10.1021/acsaelm.5c00201 (PMC12080251; doi:10.1021/acsaelm.5c00201)
Supplement: Supplementary file 1 — el5c00201_si_001.pdf [file el5c00201_si_001.pdf]

Supporting information

TITLE. Improving light stability of non-fullerene acceptor inverted organic solar cell by incorporating a mixed nanocomposite metal oxide electron transporting layer.

Apostolos Ioakeimidis [1], Fedros Galatopoulos [1], Alina Hauser [2], Michael Rossier [2], Stelios A. Choulis [1*]*

[1] Molecular Electronics and Photonics Research Unit, Department of Mechanical Engineering and Materials Science and Engineering, Cyprus University of Technology, Limassol, 3603 (Cyprus).

[2] Avantama AG, Laubisruetistr. 50, Staefa 8712, Switzerland

Keywords: Organic Photovoltaics, metal oxides, electron transporting layers, lifetime, inverted organic solar cells

Corresponding Authors

Stelios Choulis: stelios.choulis@cut.ac.cy

Apostolos Ioakeimidis: a.ioakeimidis@cut.ac.cy

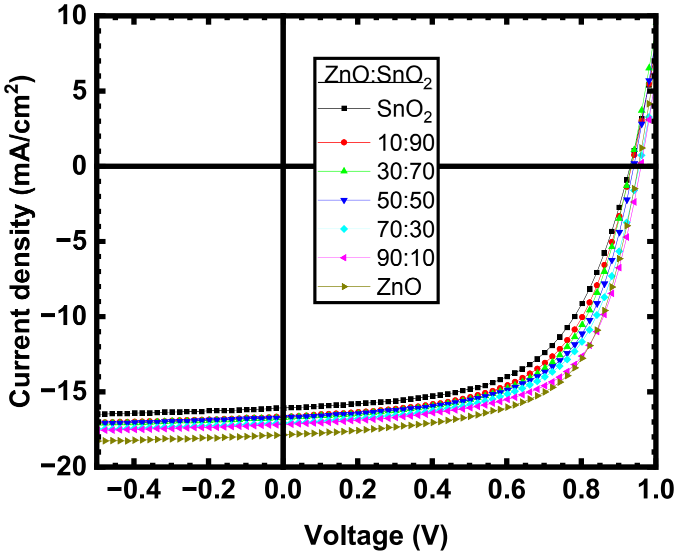

Figure S1. J-V curves of the highest PCE devices under one sun simulated light (A.M. 1.5) of the unencapsulated inverted T1:IT4F based OSC incorporating various ratios of mixed oxide ZnO:SnO_2 ETL.

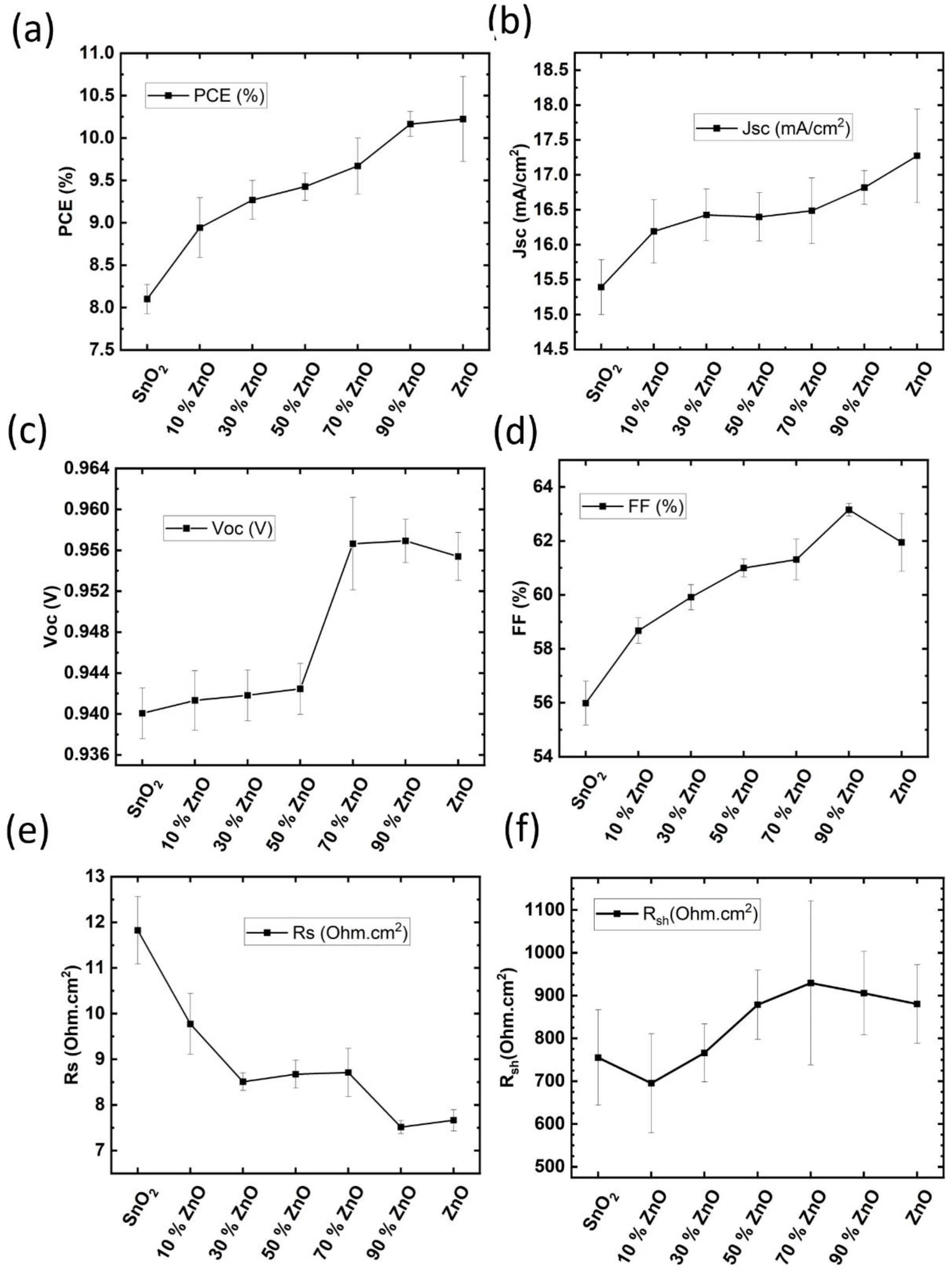

Figure S2. Mean (a) PCE, (b) Jsc, (c) Voc, (d) FF and (e) series resistance (Rs) and (f) shunt resistance (Rsh) under one sun simulated light (A.M. 1.5) of the unencapsulated inverted T1:IT4F based OSC incorporating various ratios of mixed oxide ZnO:SnO_2 ETL.

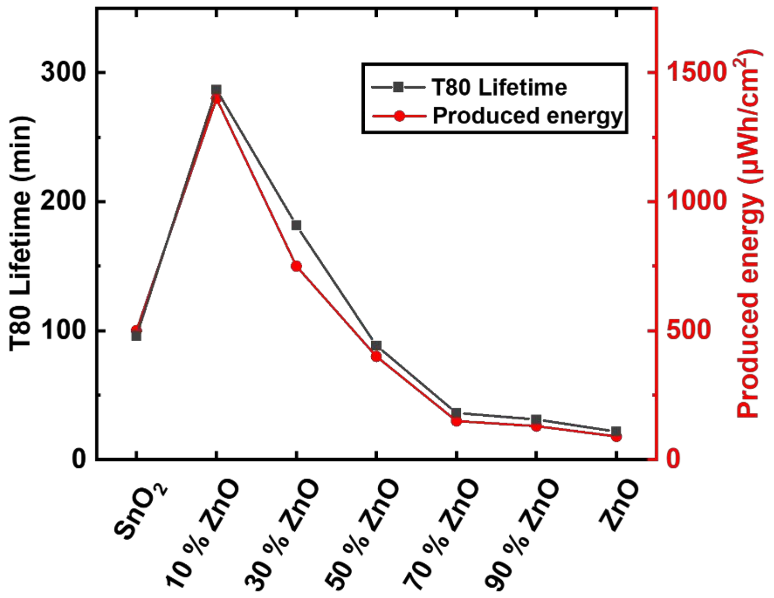

Figure S3. T80 lifetime (left axis) and the corresponding calculated produced energy (right axis) of the unencapsulated T1:IT4F based inverted OSC incorporating various ratios of mixed oxide ZnO:SnO_2 ETL

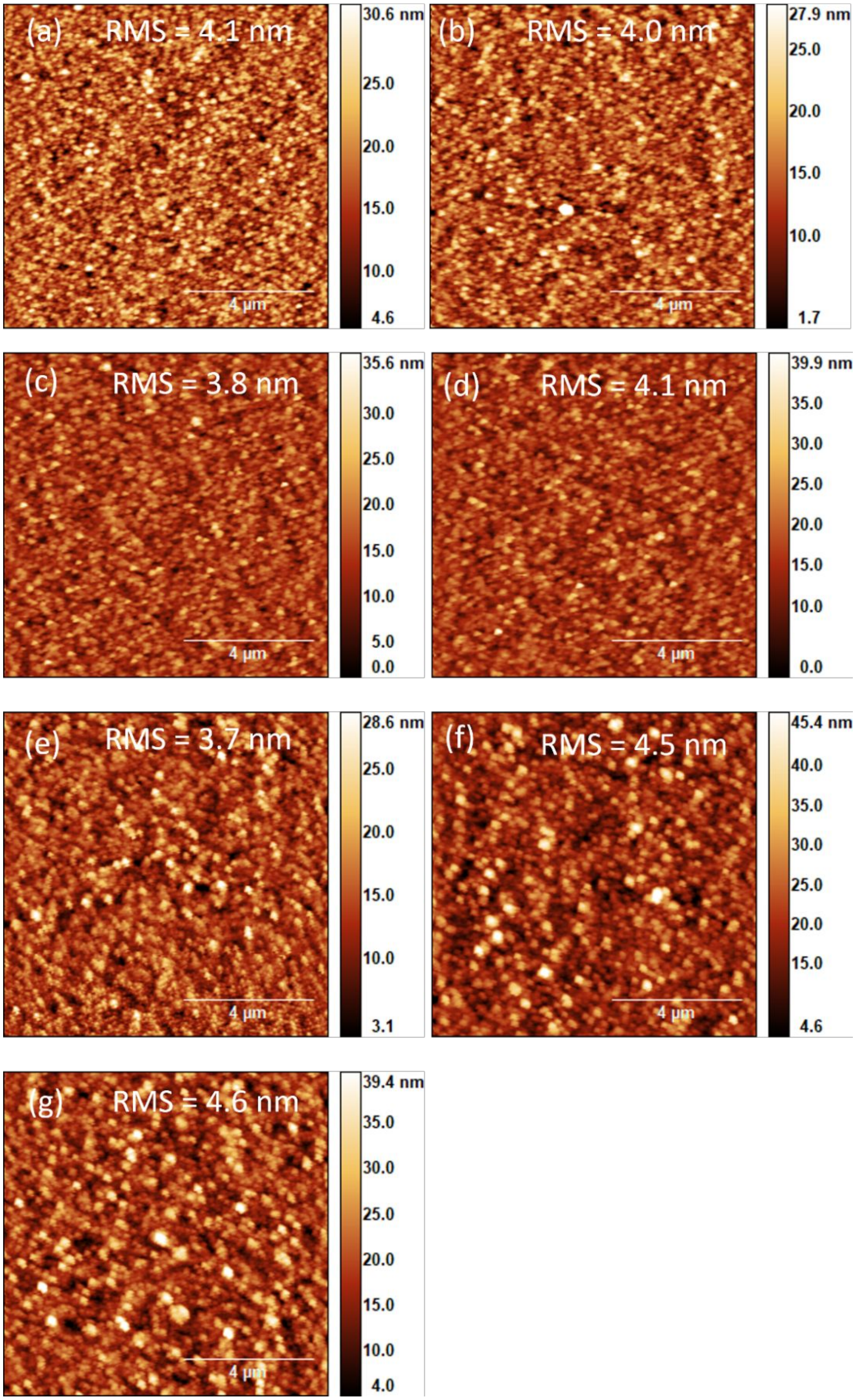

Figure S4. AFM topography images (10x10 µm) of (a) SnO_2, (b) 10 %:90 %, (c) 30%:70%, (d) 50%:50%, (e) 70%:30%, (f) 90%:10% ZnO:SnO_2 and (g) ZnO ETL fabricated on top of ITO.

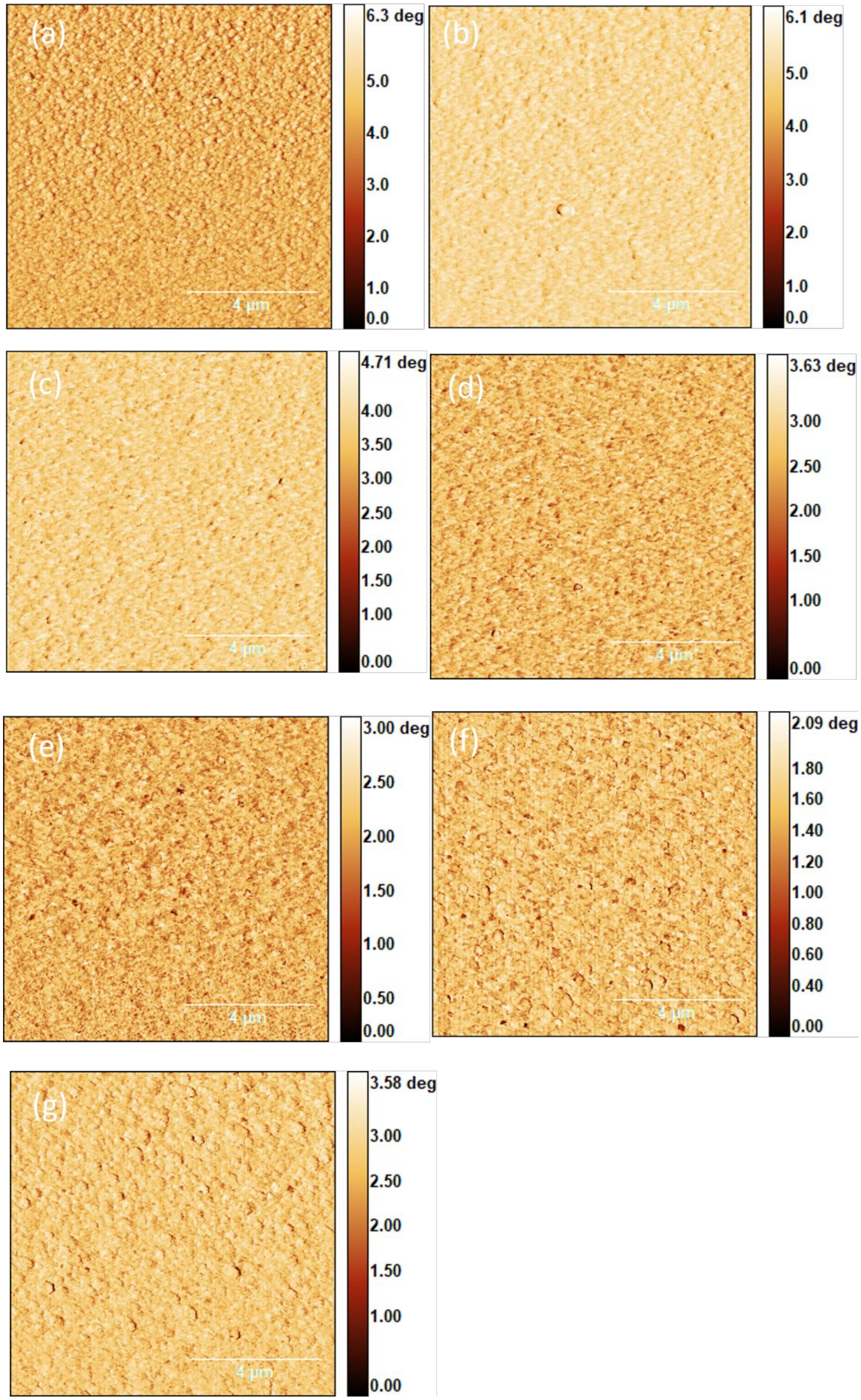

Figure S5. AFM phase images (10x10 µm) of (a) SnO_2, (b) 10 %:90 %, (c) 30%:70%, (d) 50:50%, (e) 70%:30%, (f) 90%:10% $ZnO:SnO_2$ and (g) ZnO ETL fabricated on top of ITO.

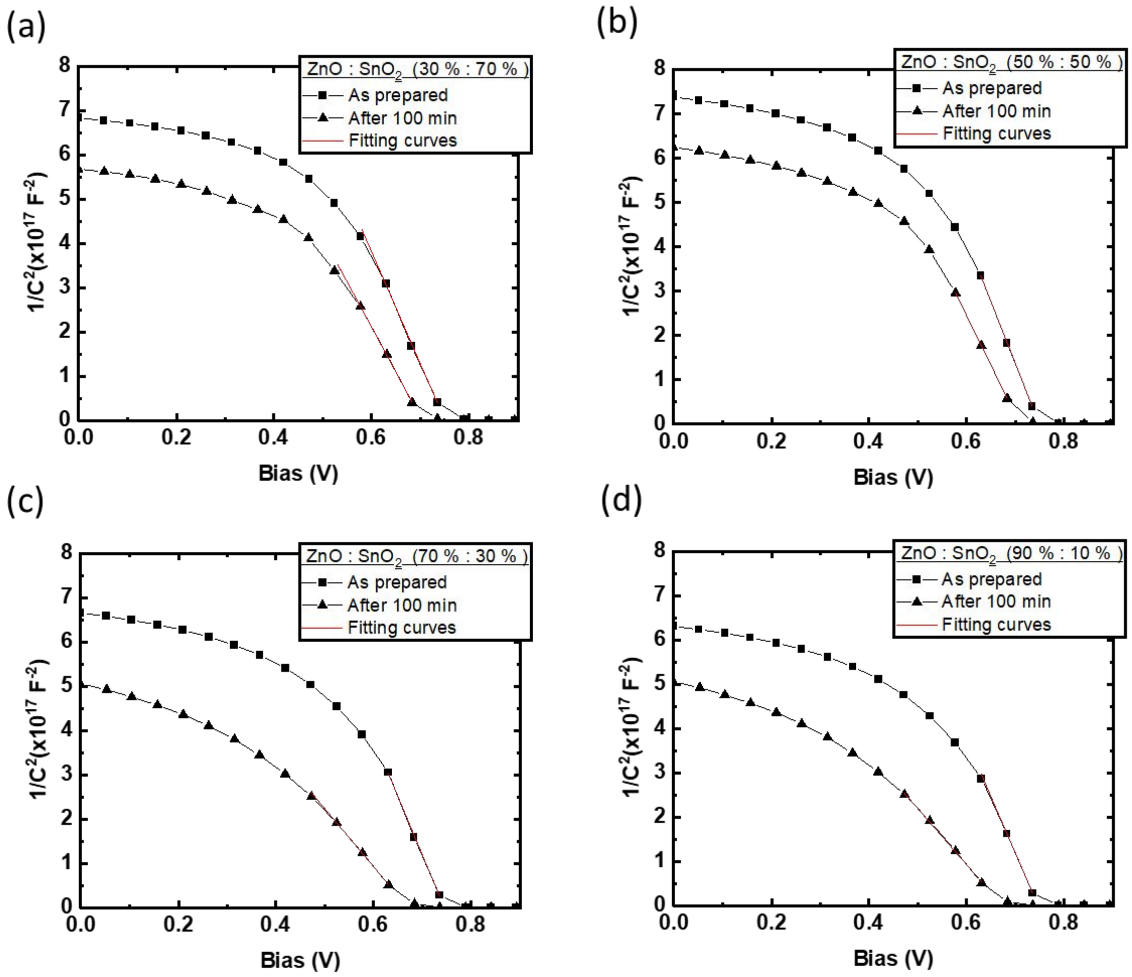

Figure S6 Mott-Schottky plot of unencapsulated T1:IT4F based inverted OSC incorporating mixed metal oxide $ZnO:SnO_2$ (a) 30%:70%,(b) 50%:50%, (c) 70%:30% and (d) 90%:10% ETL for the as prepared and after 100 min under ISOS-L2 protocol

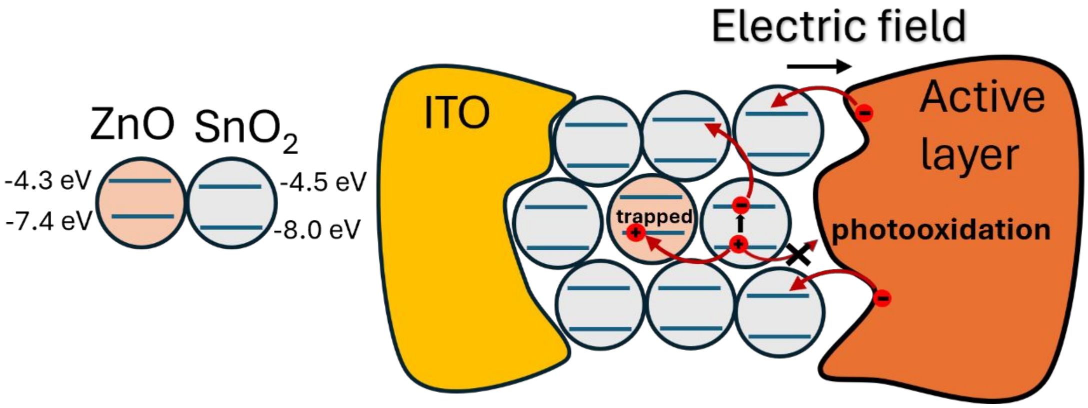

Figure S7. Schematic illustration of the restrained photooxidation at the mixed metal-oxide/ active layer interface due to the presence of a low density of ZnO nanoparticles within the SnO_2 ETL.
